# Differential Activation of pERK1/2 and c-Fos Following Injury to Different Regions of Primary Sensory Neuron

**DOI:** 10.3390/life12050752

**Published:** 2022-05-19

**Authors:** Bei Miao, Hongyu Yao, Peng Chen, Xue-Jun Song

**Affiliations:** 1 Department of Medical Neuroscience, School of Medicine, Southern University of Science and Technology, Shenzhen 518055, China; miaobei@xzhmu.edu.cn (B.M.); yaohy2020@mail.sustech.edu.cn (H.Y.); chenp6@mail.sustech.edu.cn (P.C.); 2 SUSTech Center for Pain Medicine, School of Medicine, Southern University of Science and Technology, Shenzhen 518055, China; 3 Department of Gastroenterology, Xuzhou Medical University Affiliated Hospital, Xuzhou 221004, China

**Keywords:** pERK1/2, c-Fos, partial dorsal rhizotomy (PDR), chronic compression of DRG (CCD), chronic constriction injury (CCI), axonal transport

## Abstract

Nerve injury causes hyperexcitability of the dorsal root ganglion (DRG) and spinal dorsal horn (DH) neurons, which results in neuropathic pain. We have previously demonstrated that partial dorsal rhizotomy (PDR) produced less severe pain-like behavior than chronic constriction injury (CCI) or chronic compression of DRG (CCD) and did not enhance DRG neuronal excitability. However, the mechanisms underlying such discrepancy remain unclear. This study was designed to compare the activation of phosphorylated extracellular signal-regulated kinase 1/2 (pERK1/2) in DRG and DH, and c-Fos in DH following treatments of CCI, CCD, and PDR. We confirmed that thermal hyperalgesia produced by PDR was less severe than that produced by CCI or CCD. We showed that pERK1/2 in DRG and DH was greatly activated by CCI or CCD, whereas PDR produced only transient and mild pERK1/2 activation. CCI, CCD, and PDR induced robust c-Fos expression in DH; nevertheless, c-Fos^+^ neurons following PDR were much fewer than that following CCI or CCD. Blocking retrograde axonal transport by colchicine proximal to the CCI injury site diminished thermal hyperalgesia and inhibited pERK1/2 and c-Fos activation. These findings demonstrate that less severe pain-like behavior produced by PDR than CCI or CCD attributes to less activation of pERK1/2 and c-Fos. Such neurochemical activation partially relies on retrograde axonal transport of certain “injury signals” from the peripheral injured site to DRG somata.

## 1. Introduction

Nerve injury that produces intractable persistent pain manifested as spontaneous pain, allodynia, and hyperalgesia remains a major clinical challenge. Nerve injury increases the sensitivity of DRG nociceptors and enhances synaptic plasticity between DRG and DH neurons giving rise to spinal sensitization exhibiting hyperalgesia and allodynia [1,2]. Using neuropathic pain models of chronic compression of DRG (CCD) [3,4], chronic constriction injury (CCI) [5], and partial dorsal rhizotomy (PDR) [6], we have previously demonstrated that injury to the peripheral branches of the axon (CCI) or somata (CCD) increases DRG neuron excitability and produces significant thermal hyperalgesia and mechanical allodynia. In contrast, injury to the central branches of the axon (PDR) produces neuropathic pain, which is less severe than that following CCI or CCD and does not alter DRG neuron excitability [6]. However, mechanisms underlying such discrepancy following injuries to different regions of DRG remain unknown.

The peripheral axons of the DRG can retrogradely transport injury-related proteins, including cytokines and neurotrophic factors, from injured sites to its somata [7,8,9,10]. These proteins were initially acknowledged as “injury signals” [11], which activate downstream cascades backward to DRG somata and alter gene expression and neuronal functions [11,12,13,14]. Colchicine, a microtubule depolymerizer for blocking axonal transport, can diminish the injury-induced hyperexcitability of sensory neurons [12] and attenuate behaviorally-expressed hyperalgesia [15]. Multiple studies demonstrated that pERK1/2 is important for the hyperexcitability of DRG and DH neurons during the development of chronic pain after nerve injury [16,17,18,19,20]. Responsive to upstream signaling, pERK1/2 enhances sensory neuron excitability via both transcriptional and non-transcriptional modulations [20,21,22,23,24,25,26,27]. The immediate early gene c-Fos is expressed in DH responding to noxious stimuli, similar to pERK1/2. Increased c-Fos in DH is considered a marker of central sensitization during chronic pain development [28,29,30,31,32,33]. Thus, pERK1/2 and c-Fos are the crucial neurochemicals associated with neuropathic pain.

In this study, we hypothesized that activated “injury signals” might be transported retrogradely to DRG somata in CCI and CCD, while PDR-produced “injury signals” might be only transported to central terminals rather than DRG somata. Hence, the pain-associated neurochemicals might respond differently during the development of neuropathic pain following CCI, CCD, or PDR. We showed that pERK1/2 and c-Fos activation in DRG and/or DH following PDR were significantly less than that following CCI and CCD, respectively. In the CCI model, blocking retrograde axonal transport diminished activation of pERK1/2 and c-Fos as well as thermal hyperalgesia, suggesting that peripheral “injury signals” from the injured site are critical for the hyperexcitability of DRG neurons and the development of neuropathic pain.

## 2. Materials and Methods

### 2.1. Animals, Anesthesia and Surgeries

**Figure 1:**
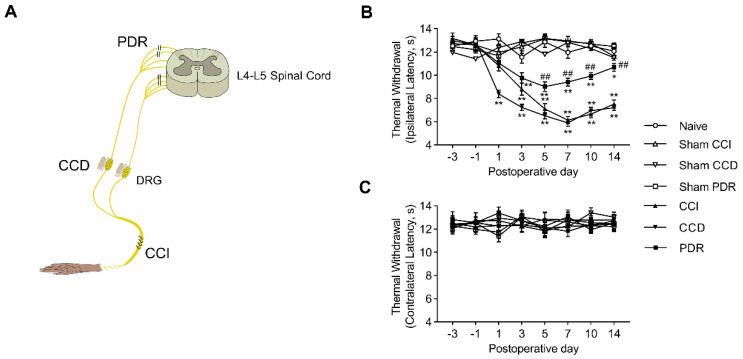
(**A**) Schematic illustration of the animal models of neuropathic pain produced by partial dorsal rhizotomy (PDR), chronic compression of dorsal root ganglion (DRG) (CCD), and chronic constriction injury of sciatic nerve (CCI), adapted with permission from Ref. [6]. Copyright 2003, Song et al. (**B**,**C**) Thermal withdrawal latency by Hargreaves test of ipsilateral (**B**) and contralateral (**C**) hind paw after CCI, CCD, PDR, and corresponding sham surgeries (*n* = 8 in each) showing less severity of behavioral−expressed thermal hyperalgesia induced by PDR than CCI or CCD. Two−way ANOVA, * *p* < 0.05, ** *p* < 0.01 versus corresponding sham groups, ## *p* < 0.01 versus CCI and CCD.

Adult male Sprague-Dawley rats (200–250 g at the beginning of the experiment) were employed in the present study. The animals were provided and housed by the Experimental Animal Center of the Southern University of Science and Technology under a 12 h/12 h light–dark cycle regime, with free access to food and water. All of the experimental protocols were approved by the Animal Care and Use Committee of the university and were in accordance with the Declaration of the National Institutes of Health Guide for Care and Use of Laboratory Animals. All of the surgeries were performed under anesthesia induced by sodium pentobarbital (40 mg/kg, i.p.). After surgery, the muscle and skin layers were sutured. Augmentin, one oral antibiotic composed of 80% Amoxicillin and 20% clavulanic acid, was then administrated in the drinking water for each rat (7.52 g in 500 mL) after surgery for 7 days. The animals were kept for up to 14 days after surgery for subsequent behavior tests, Western Blotting, and immunofluorescence analysis. The models of PDR, CCD, and CCI, are illustrated in Figure 1A. The surgical procedures are described below (PDR, CCD, CCI, sham surgery, and naive control).

PDR. The surgical procedure of PDR is the same as that we described previously [6]. In brief, under anesthesia, a midline incision was made between T12–L3 in the experimental rats. The paraspinal muscles were separated from the spinal processes on the left side. The transverse processes of L1 and L2 were exposed by scraping off the attached ligaments, and a small “window” laminectomy was performed unilaterally at L1–L2 to expose the L4 and L5 dorsal roots central to the DRGs and close to the spinal cord. The dura was opened to approximately 5 mm in length, and the L4 and L5 dorsal roots were identified under a dissecting microscope. The rootlets of L4 and L5 were carefully isolated from each other. Normally there are four rootlets within each dorsal root. The rostral half of the rootlets (2 of 4) were transected (approximately 1–2 mm), whereas the caudal half were kept intact. Procaine (2%) was dropped onto the rootlets before transection.

CCD. Chronic DRG compression was produced by surgically implanting stainless steel rods unilaterally into the intervertebral foramen at L4 and L5. The surgical procedure of CCD was the same as that described previously [3,4]. In brief, the rats were anesthetized, paraspinal muscles were separated from the mammillary and transverse processes, and the intervertebral foramina of L4 and L5 were exposed. A stainless-steel L-shaped rod, 4 mm in length and 0.6 mm in diameter, was implanted into each foramen, one at L4 and the other at L5.

CCI. The procedure of CCI was the same as that described previously [5]. Briefly, the rats were anesthetized, and the left common sciatic nerve was exposed at the middle level of the thigh. Proximal to the sciatica’s trifurcation, about 7 mm of the nerve was freed of adhering tissue, and four ligatures were tied loosely around it with about 1 mm spacing. The length of the nerve affected was about 4–5 mm long.

Sham surgery. The rats were evenly divided into three groups and received sham surgery. The surgical procedure was identical to that described in PDR, CCD, or CCI but without injury to the dorsal root, DRG, or sciatic nerve.

Naïve control. Another group of rats served as the unoperated control and did not receive any surgery or injury.

### 2.2. Administration of Colchicine

Upon the anesthetization of the rats, a small piece of Parafilm was inserted underneath the left sciatic nerve of the rat. At this site, 2 mm × 2 mm pads of Gelfoam soaked in 50 mM of colchicine solution (Col) (#C3915, Sigma, St. Louis, MO, USA) or saline were placed around the intact epineurium forming a continuous meniscus, as described in the publication [15]. CCI was then performed on the rats 20 min later. The group of rats that received Col treatment was referred to as CCI+Col, and that received saline control was referred to as CCI+Vehicle. All efforts were made to minimize animal suffering. Drug administration and surgery were invariably carried out between 10 a.m. and 15 p.m. to avoid possible diurnal rhythm effects.

### 2.3. Pain-Like Behavior Test

The Hargreaves test was applied to determine the presence of thermal hyperalgesia by measuring foot withdrawal latency to heat stimulation [34]. Each rat was placed in a box (17 × 22 × 14 cm^3^) containing a smooth glass floor. The temperature of the glass was measured and maintained at 25 °C. A heat source (IITC Model 336 Analgesia Meter, IITC Life Science, Woodland Hills, CA, USA) was focused on a portion of the hindpaw, which was flush against the glass, and a radiant thermal stimulus was delivered to that site. The stimulus shut off automatically when the hindpaw moved (or after 20 s to prevent tissue damage). The intensity of the radiant heat light source was set at 50 Units on the test equipment, which elicited approximately 9–12 s latency of paw withdrawal in the naïve control animals. This radiant heat intensity was constant throughout all experiments. Thermal stimuli were delivered six times to each hind paw at 5- to 6-min intervals. For the results expressing thermal hyperalgesia, the values are calculated as the mean in the tests. The rats were tested 1 and 3 days prior to surgery as the baseline. Postoperative tests were conducted 1, 3, 5, 7, 10, and 14 days after surgery to plot the time course of thermal hyperalgesia.

### 2.4. Western Blotting

The DRGs and DH at L4 and L5 of the rats were extracted and stored in liquid nitrogen. The tissues were homogenized in 1:10 (*w*/*v*) ice-cold homogenization buffer consisting of 50 mM of MOPS (pH 7.4), 50 mM of NaF, 20 mM of NaPPi, 20 mM of β-glycerophosphate, 1 mM of EDTA, 1 mM of EGTA, 1 mM of phenylmethylsulfonyl fluoride, 10 μg/mL of leupeptin, 10 μg/mL of aprotinin, and 10 μg/mL of pepstatin A with a Teflon-glass homogenizer followed by centrifugation at 800 g for 15 min at 4 °C, and then the supernatant was used. The samples were stored at −80 °C and were thawed only once. After the determination of the protein concentration, 50 μg of protein from each sample was heated at 100 °C for 5 min with a loading buffer containing 0.125 M Tris-HCl (pH 6.8), 20% glycerol, 4% SDS, 10% mercaptoethanol, and 0.002% bromphenol blue and separated by SDS-PAGE using 10% acrylamide gels. The proteins were electrotransferred onto a nitrocellulose filter (pore size, 0.45 μm). Blotting filters were incubated with 5% bovine serum albumin in TBST (10 mM pH 7.5 Tris, 150 mM NaCl, 0.05% Tween 20) at 4 °C for 6 h and then incubated with anti-phosphorylated ERK1/2 antibody (1:1000, Rabbit, AB_10694057, Cell Signaling Technology, Danvers, MA, USA), anti-ERK1/2 antibody (1:1000, Mouse, AB_260501, Sigma) at 4 °C overnight. The detection was carried out using HRP-conjugated anti-rabbit IgG (1:10,000, Goat, AB_2099233, Cell Signaling Technology) and HRP-conjugated anti-mouse IgG (1:10,000, Goat, AB_330924, Cell Signaling Technology). All of the antibodies were diluted in TBST containing 1% bovine serum albumin. The bands were illuminated and detected by the image processer (Tanon 5200, Tanon, Shanghai, China). The phosphorylation level of ERK1/2 was expressed as the ratio of the optical density of the pERK1/2 band to the ERK1/2 band in the same lane. The value of pERK1/2: ERK1/2 was then expressed as a fold change of sham control on the same filter.

### 2.5. Immunofluorescence

Deeply anesthetized rats were transcardially perfused with phosphate buffer saline (PBS) followed by 4% formaldehyde. L4–L5 DRGs and spinal cord segments were removed and post-fixed in 4% formaldehyde overnight. After post-fixation, antigen retrieval was conducted in a citric acid buffer. Then the tissues were transferred into 40% sucrose (in PBS) for 3 days for dehydration. The tissues were sectioned to a 30-μm thickness using the Leica CM1520 vibratome. The free-floating sections were blocked in PBS containing 10% donkey serum with 0.3% Triton X-100 for 2 h and incubated in the primary antibody at 4 °C overnight. The sections were then washed in PBS with 0.05% Triton X-100, pH 7.6 for 5 min × three times, followed by incubation in the secondary antibody at room temperature for 2 h and washing. The sections were mounted on the Superfrost/plus microscope slides (#22-037-246, Thermo Fisher Scientific, Waltham, MA, USA) and covered with an anti-fade mounting buffer for observation. The primary antibodies used included anti-c-Fos antibody (1:1000, Rabbit, AB_2106783, Santa Cruz, CA, USA) and anti-phosphorylated ERK1/2 antibody (1:400, Rabbit, AB_10694057, Cell Signaling Technology). The secondary antibody used was anti-rabbit IgG conjugated with Alexa Fluor 488 (1:500, Donkey AB_2535792, Molecular Probes, Eugene, OR, USA). All of the antibodies were diluted in PBS containing 0.1% Triton X-100 and 3% donkey serum. All of the images were captured with a Zeiss Axioplan microscope with a CCD digital camera and processed with ImageJ.

### 2.6. Statistical Analysis

Statistical analysis was completed using GraphPad Prism 8 software. All of the data were expressed as mean ± SEM. The data from the behavior tests were analyzed using two-way ANOVA, followed by post hoc Tukey tests. Western Blotting and immunofluorescence quantification were analyzed using one-way ANOVA, followed by a post hoc Dunnett test. Prior to ANOVA analysis, the homogeneity and the normality of data were tested with the Brown–Forsythe test and Shapiro–Wilk test, respectively. *p* < 0.05 was considered significant.

## 3. Results

### 3.1. PDR Produced Less Severe Behaviorally-Expressed Thermal Hyperalgesia Than CCI or CCD

We started by confirming the previous findings in animals with CCI, CCD, or PDR treatment (Figure 1A) that PDR-produced neuropathic pain was significantly less severe than that in CCI or CCD. The latency of hindpaw withdrawal to noxious heat stimulation was tested through the time course before and after surgery. Before surgery, there was no significant difference in the values of thermal withdrawal latency of both feet in groups of CCI, CCD, PDR, and the corresponding sham controls. Behaviorally-expressed thermal hyperalgesia was developed as shown in the time course of changes in rats with CCI, CCD, and PDR treatment (Figure 1B, * *p* < 0.05, ** *p* < 0.01). It is worthy to note that the severity of thermal hyperalgesia produced by PDR was significantly less than that produced by CCI or CCD (Figure 1B, ## *p* < 0.01). This result is in accordance with what we reported previously [6]. In contrast, there were no significant alterations to thermal sensitivity found in the feet contralateral to the treatment of CCI, CCD, or PDR (Figure 1C).

### 3.2. PDR Activated Less ERK1/2 Phosphorylation in DRG and DH Than CCI or CCD

**Figure 2:**
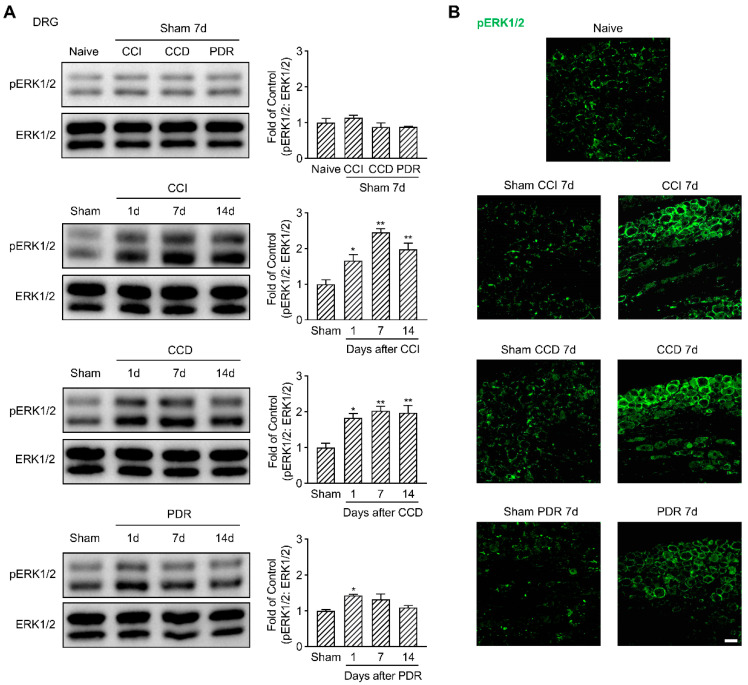
Less activation of pERK1/2 in rat DRG after PDR than CCI or CCD. (**A**) Western Blotting showing the time course of ERK1/2 phosphorylation in DRG after CCI, CCD and PDR (*n* = 3 in each). Representative bands are shown on the left; data summary is shown on the right. One−way ANOVA, * *p* < 0.05, ** *p* < 0.01 versus corresponding sham groups. (**B**) Immunofluorescence (green) showing the expression of pERK1/2 in DRG on day 7 after CCI, CCD and PDR, as well as corresponding sham groups and naïve control. Scale bar, 50 μm.

Considering the essential role of ERK1/2 phosphorylation in the sensitization of DRG and DH neurons [20,21,22,23], we evaluated the changes of pERK1/2 in DRG and DH of the rats with different modeling. In support of previously published results [17], our Western Blotting analysis showed a significant increase in pERK1/2 in DRG starting from the 1st day that peaked on the 7th day after CCI and CCD surgeries, compared to the corresponding sham groups (Figure 2A, * *p* < 0.05, ** *p* < 0.01). It was confirmed by the immunofluorescence that the fluorescence intensity of pERK1/2 on the 7th day after CCI and CCD surgeries obviously increased in both DRG neurons and surrounding satellite glial cells (Figure 2B), while in the DRG of the naïve control or sham groups, the fluorescence of pERK1/2 was majorly located in satellite glial cells [27]. It was interesting to find that PDR also triggered pERK1/2 activation in DRG as well, but such activation only occurred on the 1st day after surgery (Figure 2A, * *p* < 0.05). Although there was a trend of pERK1/2 increase in DRG on the 7th day following PDR treatment (Figure 2A,B), it did not show a statistical difference in Western Blotting analysis compared with its sham group.

**Figure 3:**
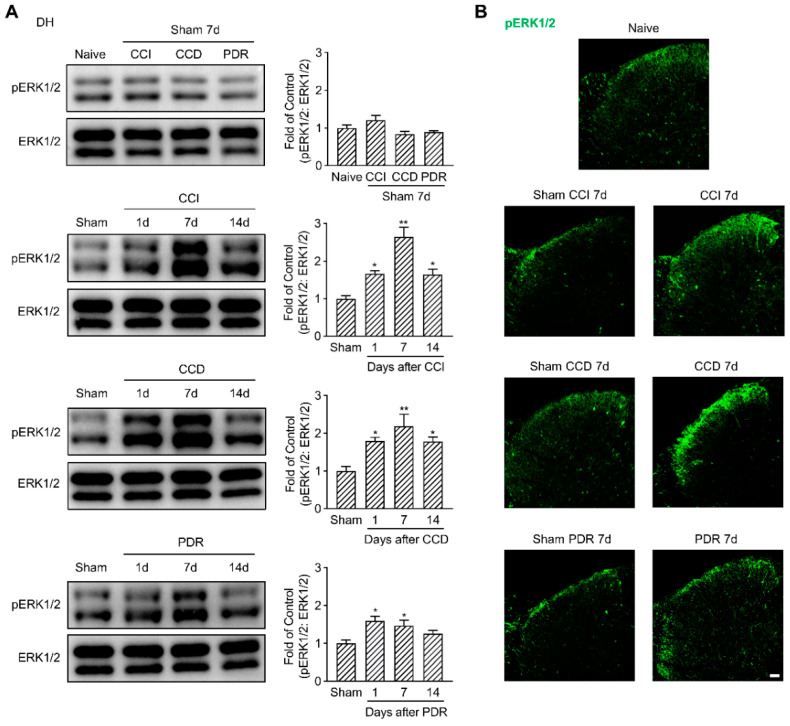
Less activation of pERK1/2 in rat DH after PDR than CCI or CCD. (**A**) Western Blotting showing the time course of ERK1/2 phosphorylation in DH after CCI, CCD, and PDR (*n* = 3 in each). Representative bands are shown on the left; data summary is shown on the right. One−way ANOVA, * *p* < 0.05, ** *p* < 0.01 versus corresponding sham groups. (**B**) Immunofluorescence (green) showing expression of pERK1/2 in DH on day 7 after CCI, CCD, and PDR, as well as corresponding sham groups and naïve control. Scale bar, 50 μm.

Parallelly, we examined ERK1/2 phosphorylation in the DH of the spinal cord. pERK1/2 was predominantly expressed in the DH neurons located in the superficial laminas, as well as the glial cells, as reported [26]. CCI and CCD surgeries dramatically induced pERK1/2 activation in DH (Figure 3A, * *p* < 0.05, ** *p* < 0.01), especially in the lamina I/II_o_ (Figure 3B). Similarly, pERK1/2 had a significant increase in DH on the 1st day and the 7th day after PDR surgery as well (Figure 3A,B, * *p* < 0.05), but PDR-induced pERK1/2 activation in DH was less than one and a half folds compared with its sham group. In contrast, the maximum fold change of pERK1/2 in DH following CCI or CCD surgery was more than two folds of their corresponding sham groups (Figure 3A). To exclude any effects on pERK1/2 activation produced by the surgical procedure, we compared each sham group with naïve control and found they did not increase pERK1/2 neither in DRG nor in DH (Figure 2 and Figure 3). Taken together, these findings provided the neurochemical basis that less pERK1/2 was activated by PDR in DRG and DH, which helps explain why PDR did not alter the excitability of DRG neurons and induced less severe thermal hyperalgesia.

### 3.3. PDR Induced Less c-Fos Expression in DH Than CCI or CCD

**Figure 4:**
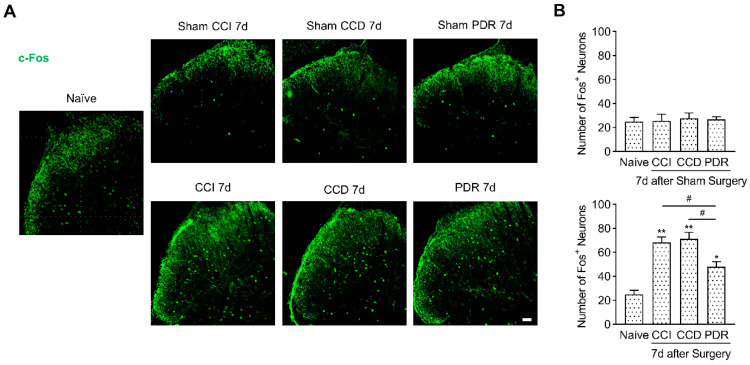
Less c-Fos expression in rat DH after PDR than CCI or CCD. (**A**) Immunofluorescence (green) showing c-Fos+ DH neurons on day 7 after CCI, CCD, PDR, corresponding sham groups and naïve control, representative confocal images are shown; (**B**) data summary of c-Fos+ neurons in (**A**), 4 slices from each individual rat used for average, *n* = 3 in each group, One−way ANOVA, * *p* < 0.05, ** *p* < 0.01 versus naive, # *p* < 0.05 versus PDR. Scale bar, 50 μm.

As the immediately early gene, c-Fos has been widely acknowledged as a biomarker of activated neurons and enhanced neural plasticity in the central nervous system [29,30]. Long-term c-Fos expression in DH was identified as a crucial indicator for central sensitization in chronic pain [28,30,31,32,33]. Next, we analyzed and compared c-Fos expression in DH after CCI, CCD, and PDR surgeries. The immunofluorescence showed that the number of DH neurons expressing c-Fos (c-Fos^+^ neurons) was significantly increased after CCI, CCD, and PDR surgeries on the 7th day (Figure 4, * *p* < 0.05, ** *p* < 0.01). Nevertheless, the increase in c-Fos^+^ neurons after PDR surgery was less than those after CCI and CCD surgeries (Figure 4, # *p* < 0.05), which was similar to the pattern of pERK1/2 activation in DH. To test whether sham surgery has a possible influence on c-Fos expression, we compared each sham group with the naïve control and did not find any significant difference in the number of c-Fos^+^ neurons among each group (Figure 4). These findings revealed that PDR surgery still caused spinal sensitization; however, it was less severe than that in CCI or CCD. Thus, our analysis of the c-Fos expression in DH supports the notion that the decreased severity of spinal sensitization is also the reason for milder thermal hyperalgesia produced by PDR than CCI or CCD.

### 3.4. Blocking Axonal Transport Suppressed CCI-Induced Thermal Hyperalgesia and Neurochemical Activations in DRG and DH

**Figure 5:**
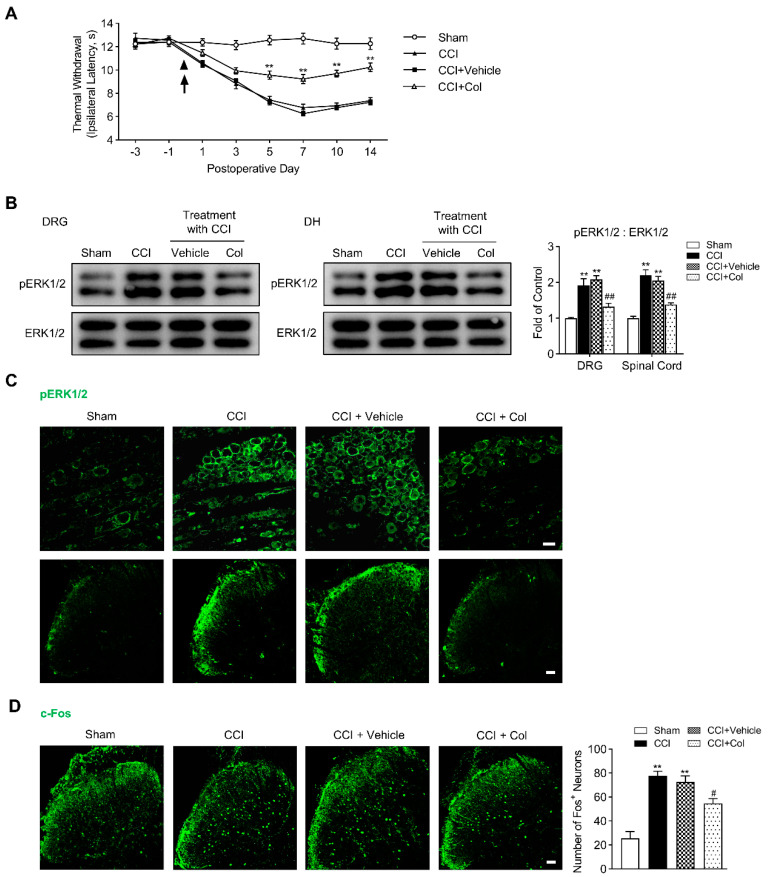
Topical application of colchicine (Col) ameliorates CCI−induced thermal hyperalgesia and suppresses underlying neurochemical alterations in DRG and DH. (**A**) Hargreaves test showing the higher thermal withdrawal latency of ipsilateral hind paw along with persistent Col treatment, two−way ANOVA, ** *p* < 0.01 versus CCI + Vehicle (*n* = 8 in each). (**B**) Western Blotting showing the inhibitory effects of Col on CCI−induced ERK1/2 phosphorylation in rat DRG and DH (*n* = 3 in each). Representative bands are shown on the left; data summary is shown on the right. One−way ANOVA, ** *p* < 0.01 versus sham group, ## *p* < 0.01 versus CCI + Vehicle. (**C**) Immunofluorescence (green) showing the inhibitory effects of Col on CCI−induced ERK1/2 phosphorylation in rat DRG (up) and DH (down). Scale bar, 50 μm. (**D**) Immunofluorescence (green) showing the inhibitory effects of Col on CCI−induced c-Fos expression in DH neurons (4 slices from each individual rat used for average, *n* = 3 in each group). Representative confocal images are shown on the left; data summary of c-Fos+ neurons is shown on the right, One−way ANOVA, ** *p* < 0.01 versus sham, # *p* < 0.05 versus CCI + Vehicle. Scale bar, 50 μm.

Why do injuries to the peripheral branches of the axon (CCI) or DRG somata (CCD) intensely activate ERK1/2 phosphorylation and increase the excitability of DRG neurons, but the injury to the central branches of axon (PDR) did not [6]? Ambron and Walters hypothesized that axonal injury unmasks nuclear localization signals in certain axonplasmic proteins at the injury site, which renders some proteins, so-called “injury signals”, to be transported retrogradely to the somata of primary sensory neurons and activate downstream transcription factors to induce the expression of early and late genes [11]. Transcriptional cascades finally lead to functional changes in primary sensory neurons [11,12,13]. The inhibitor of axonal transport was shown to block the axonal injury-induced hyperexcitability of *Aplysia* sensory neurons [12] and produce an analgesic effect on the thermal hyperalgesia evoked by the injury to the sciatic nerve in rats (CCI) [15]. Here, with pERK1/2 and c-Fos as indicators for the injury-induced neurochemical alteration, we examined if blocking retrograde axonal transport at the peripheral injury site affects behavioral-expressed hyperalgesia and associated neurochemical activations in DRG and DH after CCI treatment. In agreement with Yamamoto’s results [15], the Hargreaves test showed that persistent Col treatment partially rescued the significant decrease in thermal withdrawal latency induced by CCI (Figure 5A, ** *p* < 0.01), whereas saline, the vehicle reagent of Col, cannot produce an analgesic effect. It was clearly illuminated by Western Blotting and immunofluorescence that pERK1/2 activation in DRG and DH on the 7th day after CCI surgery was markedly inhibited by Col, but not its vehicle reagent (Figure 5B,C, ## *p* < 0.01).

Again, we continued to examine the effect on CCI-induced c-Fos expression in DH produced by Col. In vehicle-treated rats, the c-Fos expression in DH did not differ with that in the sham control. However, in the Col-treated rats, we observed a profound reduction in c-Fos^+^ neurons in DH on the 7th day after CCI (Figure 5D, # *p* < 0.05). Thus, our data indicate that pERK1/2 activation and c-Fos expression in DRG and DH after CCI surgery rely on the retrograde axonal transport from the peripheral injury site to the DRG somata, such that Col-suppressed pERK1/2 activation and c-Fos induction synergistically accounts for the diminished thermal hyperalgesia after CCI.

## 4. Discussion

Our study reveals the differential neurochemical alterations following CCI, CCD, and PDR treatments, underlying the discrepant behaviorally-expressed pain and DRG neuron excitability. The principal findings are two-fold: (1) ERK1/2 phosphorylation in DRG and DH, as well as c-Fos expression in DH after PDR treatment, are less activated than those following CCI or CCD treatment; (2) the early blocking of retrograde axonal transport with Col proximal to peripheral injured site suppresses pERK1/2 and c-Fos activation in DRG and/or DH and diminishes the thermal hyperalgesia after CCI treatment. These findings provide the neurochemical basis for the discrepant behaviorally-expressed pain in three neuropathic pain models and suggest a new therapeutic target for the treatment after peripheral nerve injury.

The results of differential pERK1/2 and c-Fos activation in DRG and DH following CCI, CCD, and PDR in our present study extend the research on the mechanisms underlying the discrepant behaviorally-expressed neuropathic pain [6]. Our previous studies have suggested that the injury to different regions of DRG neurons can induce diverse alterations to the membrane excitability and plasticity of DRG and DH neurons [6,35,36]. The major finding of this study is that PDR induces less pERK1/2 and c-Fos activation in DRG and DH, in addition to producing less severe neuropathic pain compared with CCI and CCD. The activity-dependent pERK1/2 activation in DRG and DH neurons is kept closely connected with their excitability and plasticity during neuropathic pain [20,21,22,23]. Here, we demonstrate that pERK1/2 activation in DRG and DH is transient and mild following PDR. In contrast, pERK1/2 activation is prolonged and robust following CCI and CCD. These results help explain why PDR did not increase the excitability of DRG neurons, unlike CCI or CCD, and why PDR produced less severe thermal hyperalgesia than CCI or CCD [6]. Despite some differences in the time course of expression, cellular, and subcellular location from pERK1/2 [28], c-Fos is also a cellular marker for neuronal activation in DH [29,30,31,32,33]. We illustrate that the c-Fos^+^ neurons in DH following PDR are significantly fewer than that following CCI or CCD, which indicates milder spinal central sensitization following PDR compared with CCI or CCD. Thus, the results of pERK1/2 and c-Fos activation could explain and provide the neural basis for the less severe behaviorally-expressed pain produced by PDR.

The reason why PDR activated less pERK1/2 and c-Fos than CCI or CCD was then investigated in this study. According to Ambron and Walters, certain axonplasmic proteins, called “injury signals”, are transported retrogradely along axons, from the peripheral injured site to the somata of primary sensory neurons after a peripheral nerve injury [11]. These “injury signals” activate downstream kinases or transcription factors [11,12,13,14], which are integrated by primary sensory neurons that determine the changes in the function or the expression of neuropeptides, receptors, and ion channels [37,38,39,40]. Such injury signals are, for example, nerve growth factor (NGF) and TrkA receptor [8,23], P2X3 receptors [41], and cytokines [7,9,10], which can be retrogradely transported and activated downstream MAPK cascades such as pERK1/2 and cAMP-response element-binding protein (CREB) in DRG somata [9]. Since the injured sites in CCI, CCD, and PDR are anatomically distinct, we hereby suppose that CCI activates these “injury signals” that were transported retrogradely to the DRG somata; however, PDR activates the “injury signals” in the dorsal root that were mainly transported to the central terminals in DH rather than to the DRG somata. We demonstrated that blocking axonal transport with colchicine proximal to the CCI injury site not only reduces pERK1/2 and c-Fos activation but also produces a long-lasting analgesic effect on CCI-induced thermal hyperalgesia, which agrees with Yamamoto’s results [15]. With pERK1/2 and c-Fos, our data provide new evidence to support the idea that the retrograde axonal transport of “injury signals” from a peripheral injured site to the DRG somata is crucial for DRG sensitization in pain development. The limitation here in this study is that the surgical procedure of colchicine treatment is not technically applicable to PDR surgery; therefore, we did not test if the proximal application of colchicine to the PDR injury site can attenuate PDR-induced hyperalgesia. However, our previous data illustrated that PDR-induced hyperalgesia and spinal central sensitization are dependent on NMDA receptors in DH, which suggests that the injury signals in the dorsal roots may be transported to central terminals and enhance DH neuron excitability via NMDA receptors [6].

Clinical approaches to treat chronic neuropathic pain are quite limited and insufficient due to a lack of specific cellular and molecular targets. In most cases, surgery is involved in treating neuropathic pain, such as dorsal root entry zone (DREZ) lesioning (or DREZotomy). DREZotomy, which PDR simulates, is an effective way to treat intractable chronic pain, including cancer pain [42], brachial plexus avulsion pain [43], etc. Our results suggest that though DREZotomy still causes pain, its painful intensity is not comparable with the chronic neuropathic pain mentioned above, as pERK1/2 and c-Fos in DRG and DH are less activated by DREZotomy. Additionally, DREZotomy diminishes the signal transduction from the sensitized DRG neurons to the DH neurons, which together explains why DREZotomy is helpful in treating chronic pain. Despite surgical strategies such as DREZotomy, pharmacological approaches for treating neuropathic pain are lacking. The clinical analgesics targeting DRG neurons, such as pregabalin [44] or procaine [45], which inhibit voltage-gated calcium channels or sodium channels, can produce effective but not long-lasting analgesia, as they are only able to suppress the activity but not the sensitization process of DRG neurons. Different from pregabalin or procaine, axonal transport blockade produces durable analgesia in the CCI model. Our findings in this study, together with the previous report [15], demonstrate that the axonal transport of injury-related proteins in the peripheral axons of DRG neurons plays an essential role in DRG neuron sensitization during the development of neuropathic pain. As shown in this study, the retrograde axonal transport of such signals leads to pERK1/2 activation in the somata of DRG neurons and the subsequent pERK1/2 and c-Fos activation in the DH neurons. Our results suggest that the early blockade of the axonal transport proximal to the peripheral injured sites may be an effective pharmacological approach for treating neuropathic pain.

## Data Availability

Not applicable.
